# A New Measurement Technique of the Characteristics of Nutrient Artery Canals in Tibias Using Materialise's Interactive Medical Image Control System Software

**DOI:** 10.1155/2015/171672

**Published:** 2015-12-15

**Authors:** Jiantao Li, Hao Zhang, Peng Yin, Xiuyun Su, Zhe Zhao, Jianfeng Zhou, Chen Li, Zhirui Li, Lihai Zhang, Peifu Tang

**Affiliations:** ^1^Department of Orthopaedics, Chinese PLA General Hospital, No. 28 Fuxing Road, Beijing 100853, China; ^2^Department of Orthopaedics, People's Liberation Army 264 Hospital, No. 30 Qiaodong Road, Taiyuan 030001, China; ^3^Medical College, Nankai University, No. 94 Weijin Road, Tianjin 300071, China; ^4^Department of Orthopaedics, The 307th Hospital of Chinese People's Liberation Army, No. 8 Dongda Road, Beijing 100071, China; ^5^Department of Orthopaedics, Beijing Tsinghua Changgung Hospital, No. 168 Litang Road, Beijing 100084, China; ^6^Department of Orthopaedics, Tianjin Hospital, No. 406 Jiefang Road, Tianjin 300211, China

## Abstract

We established a novel measurement technique to evaluate the anatomic information of nutrient artery canals using Mimics (Materialise's Interactive Medical Image Control System) software, which will provide full knowledge of nutrient artery canals to assist in the diagnosis of longitudinal fractures of tibia and choosing an optimal therapy. Here we collected Digital Imaging and Communications in Medicine (DICOM) format of 199 patients hospitalized in our hospital. All three-dimensional models of tibia in Mimics were reconstructed. In 3-matic software, we marked five points in tibia which located at intercondylar eminence, tibia tuberosity, outer ostium, inner ostium, and bottom of medial malleolus. We then recorded *Z*-coordinates values of the five points and performed statistical analysis. Our results indicate that foramen was found to be absent in 9 (2.3%) tibias, and 379 (95.2%) tibias had single nutrient foramen. The double foramina was observed in 10 (2.5%) tibias. The mean of tibia length was 358 ± 22 mm. The mean foraminal index was 31.8%  ± 3%. The mean distance between tibial tuberosity and foramen (TFD) is 66 ± 12 mm. Foraminal index has significant positive correlation with TFD (*r* = 0.721, *P* < 0.01). Length of nutrient artery canals has significant negative correlation with TFD (*r* = −0.340, *P* < 0.01) and has significant negative correlation with foraminal index (*r* = −0.541, *P* < 0.01).

## 1. Introduction

Longitudinal stress fracture is an uncommon fracture and has been characterized as a benign, normally self-limiting condition [1–8]. The fracture could easily be misdiagnosed as infection, periostitis, or tumor due to extensive marrow edema, periosteal reaction, and new bone formation [9, 10]. Tibia is usually involved in the fractures, and the fractures were located superomedially to the nutrient foramen of the tibia [9, 11]. The position of the fracture relative to the nutrient foramen can be used as a sign to confirm the diagnosis [9]. Therefore, to investigate and identify the locations of nutrient foramina is very important for diagnosing the longitudinal stress fracture and choosing an optimal therapy.

Recently, some researchers have made substantial progress in seeking the foramina and the canals by measuring them in cadaver bone with naked eyes [11, 12], through direct radiography [13] or radiographic imaging after barium sulfate injection [14]. However, these methods are not very sensitive to ensure the details of the location. Although there is higher sensitivity by measuring nutrient foramina of femur through multidetector computed tomography [15], all these data were obtained through the two-dimensional images. Moreover, these measurements were carried out in cadaver bones and thus the results cannot be very accurate because of the bone abrading. In addition, important information of the specimen including age and gender has not been taken into account.

We present here a new three-dimensional visualizing technique of observing and measuring nutrient artery canals in tibias with the application of Materialise's Interactive Medical Image Control System (Mimics). This technique can generate more accurate location and precise three-dimensional reconstruction and can be used to analyze a large sample size to assess variations in a time-, space-, and cost-efficient manner.

Mimics software is an interactive tool for rapid prototyping, simulation modeling, and finite element analysis based on the database of computed tomographic images, magnetic resonance images, and three-dimensional rendering of objects [16–18]. It is an image-processing package with three-dimensional visualization functions that interfaces with all common scanner formats. Here we aim to use Mimics to evaluate the number, courses, location of nutrient foramina, and length of canals about tibia in order to provide full knowledge of nutrient foramina and assist in the diagnosis of longitudinal fractures of tibia and choosing an optimal therapy.

## 2. Material and Methods

Digital Imaging and Communications in Medicine (DICOM) formats were collected and transformed to the computed tomographic data and then imported to a personal computer. The DICOM data was extracted from 207 patients on whom computed tomography angiography was performed (Siemens Sensation Open 128-slice CT scanner; Siemens, Erlangen, Germany). Power settings were typically 100 kV and 105 mA, 750 ms rotation time with a slice thickness of 1.5 mm. The field of view was contained 512 × 512 pixels and increments of 1.2 mm, using detector collimation of 128 × 0.625 mm (pitch 0.933). All the patients had been hospitalized in our hospital through March 2013 to May 2014. We excluded 2 cases of tibial fracture, 3 cases of severe osteoarthritis, and 3 cases with incomplete data. Thus, a total of 398 tibias from 199 cases were included in our study. They are 135 males and 64 females. The mean age is 66.2 years, ranging from 15 to 85 years. The mean height is 1.66 m, ranging from 1.40 m to 1.84 m.

**Figure 1:**
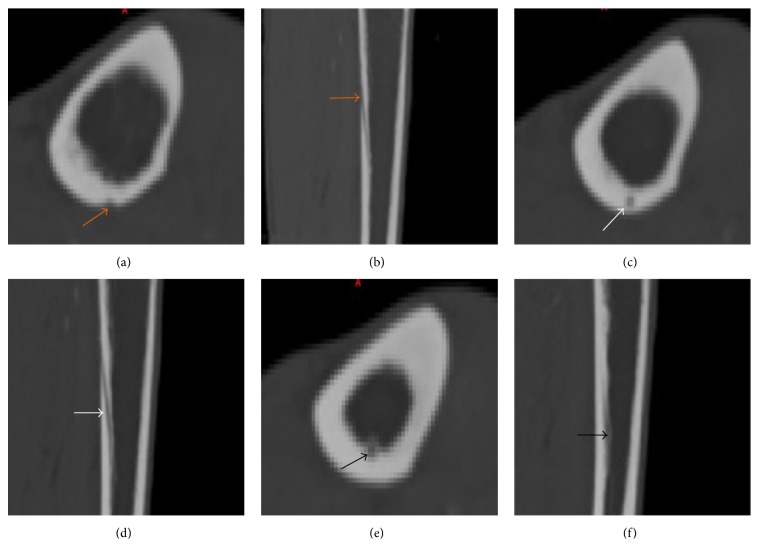
Nutrient canal showed in the axial and sagittal images (a–f) from proximal to distal. (a and b) The ostium of nutrient artery canal on the outer cortex (orange arrows), (c and d) the course through the cortex (white arrows), and (e and f) the inner ostium (black arrows).

**Figure 2:**
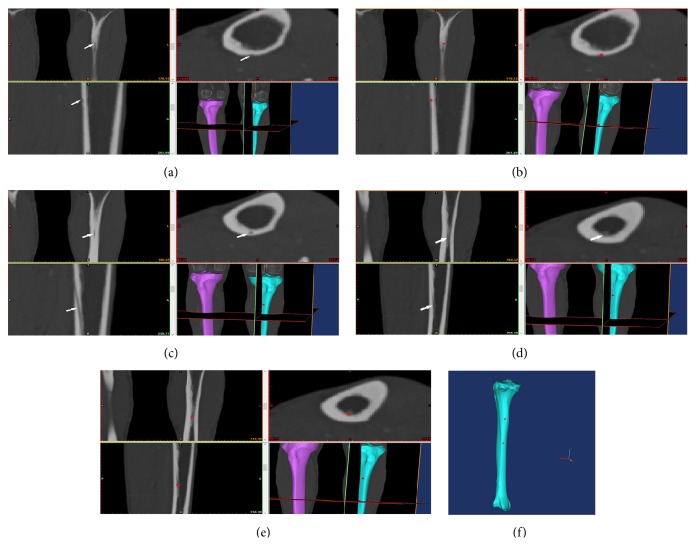
Determination of the outer ostium and inner ostium.

**Figure 3:**
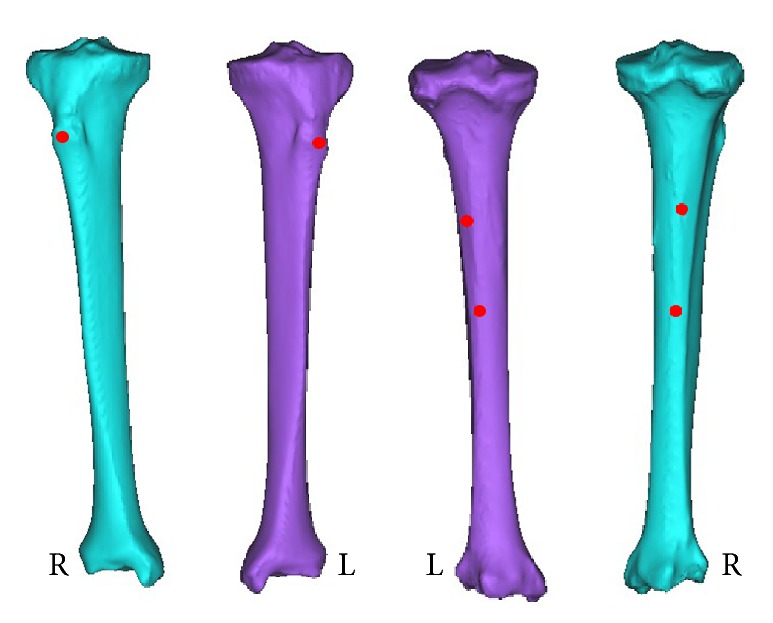
The mark of the points of tibia tuberosity, the outer ostium, and inner ostium. The left picture of the two bones is from anterior viewing, and the right is from posterior viewing.

The consecutive CT data were imported to Materialise's Interactive Medical Image Control System 10.0 (Materialise, Leuven, Belgium). We reconstructed all three-dimensional models of tibia through the protocols of thresholding, region growing, masks editing, polylines calculation, cavity filling from polylines, and so forth. The threshold units were 226 to 3071. Because the lead-filled arteries of lower extremities have a density similar to the bone, further manipulation was made to physically and digitally remove these structures to improve the clarity of the bone. Through region growing, we can make it possible to split the segmentation into what we want and also remove the flying pixels. Through polylines calculation, we can edit the details of tibia easily. After the procedures above, a three-dimensional reconstruction of the tibia bone that could be viewed stereoscopically was generated. Through browsing the axial images, coronal images, and sagittal images, we could mark the point of every tibia tuberosity, the ostium of nutrient artery canal on the outer cortex, and the inner ostium using the function of MedCAD module. A hypodense line was showed in images, which is nutrient canal. The following aspects were characterized: (1) an outer ostium on the outer cortex, (2) an uninterrupted course through the cortex, and (3) an inner ostium opening to the medullary cavity on the inner surface of the cortex (Figure 1). The determination was carried out through the following steps. Firstly, we found the outer ostium in tibia shaft, characterized by a hypodense point on the outer cortex (Figure 2(a)). Then, we marked it (Figure 2(b)). Then, we browsed the axial images primarily, and the hypodense point could be seen moving inward to the medullary cavity on the cortex. A hypodense line could be seen in coronal and sagittal images (Figure 2(c)). Thirdly, as we were browsing the axial images, an inner ostium could be found on the inner surface of the cortex, which was opening to the medullary cavity (Figure 2(d)). Combined with the coronal and sagittal images, we finally located it (Figure 2(e)). We can see the point marked on the 3D reconstructed tibia (Figure 2(f)). Under Mimics software, we marked the points of tibia tuberosity, the ostia of nutrient artery canal on the surface of outer cortex and inner cortex (Figure 3).

**Figure 4:**
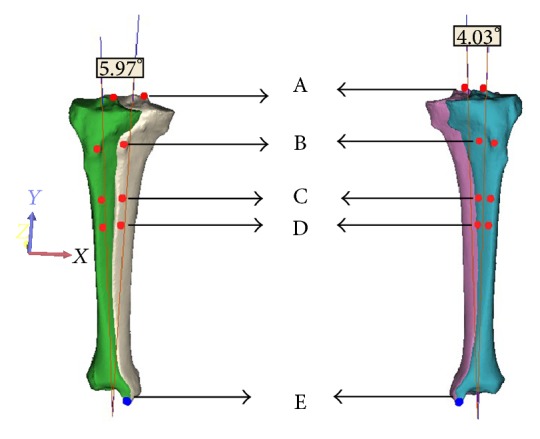
Under 3-matic software, we rotated the tibia to the standard position.

We imported the reconstruction of three-dimensional models into 3-matic software (Materialise, Leuven, Belgium) which is attached to Mimics software. We created a line that fitted the vertical axis of tibia. Then, we created another line whose direction was *z*-axis of the world coordinate system in the software and origin was the lower point of the former line. We rotated every tibia at the angle between the two lines to the standard place. Then, the intercondylar eminence of the tibia and the bottom point of medial malleolus were marked. Together with the previous record of three points, we finally got five points. From proximal to distal, they were named A, B, C, D, and E. We recorded the *Z*-coordinate values of the five points (Figure 4).

We then calculated the data and got the variables like the tibia length, foraminal index, the length of nutrient artery canal, and so forth. (Note: if double nutrient foramina were exited in one tibia, we marked the deputy nutrient foramina and recorded the data of it.)

We got the following parameters through calculating the coordinates of each point. Only *Z*-coordinates values were calculated, measured in millimeter.

 Tibial length (TL) = *Z*A–*Z*E.
 Distance between intercondylar eminence and the nutrient foramina (DIF) = *Z*A–*Z*C.
 Distance between tibial tuberosity and foramina (TFD) = *Z*B-*Z*C.
 Length of nutrient artery canals = *Z*C-*Z*D.
 Foraminal index = DIF/TL.

Statistical analysis was performed by SPSS for Windows V.13.0. The data were presented as mean ± standard deviation. Comparison between two sets of variables was performed using Student's *t*-test. The data analysis was performed using Pearson's test. Values for *P* < 0.05 were regarded as statistically significant.

## 3. Result

The cases which contain only one nutrient foramen in both of the tibias were defined as the single nutrient foramen group. Those who contain two nutrient foramina in one or both of tibias were defined as the double nutrient foramina group. We, respectively, compared the entire datum about the two groups and analyzed the data using SPSS. According to the CT photos, we named the larger pore as primary pore and the smaller pore as vice pore in the double nutrient foramina group.

Totally 398 tibias of 199 cases were included in our study. The distribution of nutrient foramina and the population characteristics are summarized in Tables 1 and 2. The bilateral nutrient foramina distribution was shown in Table 3. The mean of tibia length was 358 ± 22 mm (minimum was 295 mm and maximum was 431 mm). The mean foraminal index was 31.8%  ± 3% (minimum was 24.5% and maximum was 42.9%). The foraminal index, length of nutrient artery canals, and TFD (distance between tibial tuberosity and foramen) were showed in Table 4.

Both the length of nutrient artery canals and TFD have significant negative correlation with age (*r* = −0.288, *P* < 0.01 and *r* = −0.133,  *P* < 0.01). Foraminal index has no correlation with age but had significant positive correlation with TFD (*r* = 0.721, *P* < 0.01). Length of nutrient artery canals has significant negative correlation with TFD (*r* = −0.340, *P* < 0.01) and has significant negative correlation with foraminal index (*r* = −0.541, *P* < 0.01).

We define TFD (mm) as dependent variable (*Y*) and height (m) as independent variable (*X*). Then, we generated the regression equation which was *Y* = 21 + 30*X* (*r* = 0.188, *P* < 0.01). Length of nutrient artery canals (mm) was defined as dependent variable (*Y*) and height (m) as the independent variable (*X*). Then, the regression equation was *Y* = 65*X* − 62 (*r* = 0.377, *P* < 0.01).

## 4. Discussion

In this large sample study, we adopted a new measurement to reveal the characteristics of tibial foramina in a three-dimensional model reconstructed by Mimics. We for the first time proposed the concept of TFD, which was defined as the vertical distance between tibial tuberosity and foramina. Combination of TFD and length of nutrient artery canal can be helpful to evaluate the lower extremity trauma in clinic and prevent the damage in nutrient artery from taking inner fixation or external fixation when dealing with tibia fracture. Moreover, we provided two regression models to estimate the relationship between height and TFD or length of nutrient artery canals, which are easier to calculate the location of foramina. In most previous studies, nutrient foramina were evaluated by their outer orifices macroscopically or direct radiography [11–13, 15, 19–22]. Earlier researchers gained anatomical information through cadavers which could be inevitably frayed and thus the accuracy of data could be affected. In the present research, we obtained topographic findings of the nutrient foramina using computed tomography imaging. We further applied Mimics software to reconstruct the three-dimensional model of the tibia based on the DICOM data. The thickness of 1.5 mm greatly guaranteed the three-dimensional reconstruction quality. In addition, DICOM included the sound medical information about patients, which could be convenient for us to take it into account.

The clinical presentation of longitudinal stress fracture of the tibia (LSFT) can be misdiagnosed for osseous tumor or osteomyelitis. Open biopsy is not necessary, because false positive diagnosis of malignancy could be made due to the immature cells and osteoid tissues which are related to the healing process. In addition, biopsy itself can also increase the healing time of patients [1, 3, 9, 10, 23]. The key of diagnosing the LSFT is to find the cleft in the cortex on one or several CT or MRI images [9]. Because the abnormal MR marrow signals are nonspecific and could result from tumor or infection and also the fracture line in cortex cannot be detected efficiently [24], CT is advocated for its cost-effectiveness [3, 7, 10, 25, 26]. Most LSFT was located superomedially to the nutrient foramen of the tibia [9, 24]. The precise location in nutrient foramina of tibias can help to find the fracture line of LSFT. And the characteristics of the nutrient foramina and canals described before can help us to differentiate the fracture line easily from nutrient foramina on axial and sagittal scans of CT. In addition, the accurate knowledge of the location of the nutrient foramina in long bones can help to prevent intraoperative injuries in orthopaedic as well as in plastic and reconstructive surgery. Nutrient artery canals may be incorrectly defined as fracture lines by those who are unfamiliar with their appearances and location. Our study will provide useful information about their locations and numbers.

It was reported that 95% of the tibias had one foramen and 5% of the cases had double foramina. In some other studies, the percentage of single foramen and double foramen existing in the cases was 98.6% and 1.4%, respectively [19]. Investigations performed in China also observed similar results that 97.5% of the cases presented single foramen and 2.5% presented double foramen [27]. In the present study, we observed that foramen was found to be absent in 9 (2.3%) tibias and 379 (95.2%) tibias had single nutrient foramen. The double foramen was observed in 10 (2.5%) tibias. Our analysis suggests that the location of nutrient foramina can be determined by calculating a foraminal index (*I*) using the following formula: *I* = DNF/TL × 100%, where DNF is the distance from the proximal end of the bone to the nutrient foramina and TL is the total bone length [11]. We found that the range of the foraminal index was from 24.5% to 42.9% and the mean value was 31.8%. It should be mentioned that Prashanth et al. [19], Imre et al. [15], and Sendemir and Cimen [28] reported that the range of *I* was 28–54%, 11.0–67.2%, and 30–40%, respectively. In Prashanth et al.'s research [19], they concluded that the location of the foramina would change with aging. And in the present study, we found that there was negative correlation between the length of nutrient artery canal and age, as well as TFD and age. In addition, there is no correlation between foraminal index and age. However, the underlying mechanisms are not clear and need to be discussed in the future studies.

In this study, attention should be paid to some important aspects. (1) Smoothing and wrapped functions should not be used excessively because these functions could change the details of anatomical confirmation. (2) After model reconstruction, each tibia should be corrected to the standard position from which we could uniform the measurement criteria and also exclude the disturbances from different positions of all different tibias. (3) MedCAD module in Mimics is designed to make a bridge between medical imaging (CT and MR) and computer aided design (CAD), through which we can mark the three points of tibia.

Here, we described a new measurement method of the characteristics of nutrient foramina in tibias using Mimics. This method can be easily applied to large sample and can produce precise results through quantifying the correlation and calculating the regression equation. Most importantly, considering superficial and protruding part of tibial tuberosity in the upper tibia, we firstly proposed TFD. Through calculating the *Z*-coordinates values of the two points, it is easy and precise to measure the anatomical information. With the assist of the regression equation on TFD and height, it makes eminent sense to locate the nutrient foramina in clinical work. It should be noted that in this study we did not measure the diameters of foramina, because the edges of the hole on normal CT photograph are not easily defined. Even though high resolution- (HR-) CT scan could provide enough clarity to measure the morphology like foramen diameter, extended use of HR-CT is limited due to its high price. In addition, our sample size seems to be unitary when multiethnic condition in China is taken into account.

## 5. Conclusions

In conclusion, our study has presented a new measurement technique of the characteristics of nutrient artery canals in tibias using Mimics software, and this could provide a better understanding of the particular anatomical structure, which may be useful in various clinical implication such as longitudinal stress fractures of tibia (LSFT) and radiologic evaluation for the fracture lines.

## Figures and Tables

**Table 1:** 

Number of nutrient foramina	Tibia number		
	Left side	Right side	Total
0	3	6	9 (2.26%)
1	188	191	379 (95.23%)
2	8	2	10 (2.51%)
Total	199	199	398 (100%)

**Table 2:** 

Number of nutrient foramina	Tibia number		
	Male	Female	Total
0	0	9	9
1	263	116	379
2	7	3	10
Total	270	128	398

**Table 3:** 

Distribution of double nutrient foramina		
Left side	Right side	Total
One	Two	7 (male)
Two	One	1 (female)
Two	Two	1 (female)

**Table 4:** 

	Tibia with one foramen	Tibia with two foramina	
		Primary foramen	Vice foramen
Foraminal index	30.3% ± 2%	30.1% ± 3%	28.2% ± 7%
Length of nutrient artery canals	51 ± 9	41 ± 10	26 ± 11
TFD	66 ± 12	66 ± 15	60 ± 29
